# Functional Study of *BpPP2C1* Revealed Its Role in Salt Stress in *Betula platyphylla*

**DOI:** 10.3389/fpls.2020.617635

**Published:** 2021-01-14

**Authors:** Baoyue Xing, Chenrui Gu, Tianxu Zhang, Qingzhu Zhang, Qibin Yu, Jing Jiang, Guifeng Liu

**Affiliations:** ^1^State Key Laboratory of Tree Genetics and Breeding, Northeast Forestry University, Harbin, China; ^2^Citrus Research and Education Center, University of Florida, Lake Alfred, FL, United States

**Keywords:** *Betula platyphylla*, birch, transcriptome, salt tolerance, Cas9/gRNA, CRISPR, protein phosphatase 2C

## Abstract

PP2C protein phosphatase family is one of the largest gene families in the plant genome. Many PP2C family members are involved in the regulation of abiotic stress. We found that *BpPP2C1* gene has highly up-regulated in root under salt stress in *Betula platyphylla*. Thus, transgenic plants of *Betula platyphylla* with overexpression and knockout of *BpPP2C1* gene were generated using a zygote transformation system. Under NaCl stress treatment, we measured the phenotypic traits of transgenic plants, chlorophyll-fluorescence parameters, peroxidase (POD) activity, superoxide dismutase (SOD) activity, and malondialdehyde (MDA) content. We found that *BpPP2C1* overexpressed lines showed obvious salt tolerance, while *BpPP2C1* knocked out plants were sensitive to salt stress. Transcriptome analysis identified significantly amount of differentially expressed genes associated with salt stress in *BpPP2C1* transgenic lines, especially genes in abscisic acid signaling pathway, flavonoid biosynthetic pathway, oxidative stress and anion transport. Functional study of *BpPP2C1* in *Betula platyphylla* revealed its role in salt stress.

## Introduction

Protein phosphatase 2C (PP2C) is a branch of protein phosphatase (Protein phosphatase, PP), encoded by PP2C gene family which is one of the largest gene families in plants (Xue et al., 2008). Phosphorylation and dephosphorylation of regulatory proteins and enzymes are general mechanisms to transmit signals from the extracellular environment to the interior of the cell (Xiu-Yun and Tian, 2017). Protein phosphatases counteract the action of the protein kinases, catalyze the dephosphorylation of phosphorylated protein molecules, play an important role in the modulation and reversibility of the phosphoregulatory mechanism (Rodriguez, 1998).

PP2C is widely involved in a variety of signaling pathways in plants, including ABA regulated stress acclimation, plant growth and development, and disease resistance (Sang-Kee et al., 2006; Sari and Palva, 2010; Carrasco et al., 2014). The protein phosphatase 2C (PP2C) family in the *Arabidopsis* genome contained 80 members are divided into 13 subfamilies (A-H) (Xue et al., 2008). *AtPP2CA* belongs to subfamily A of *Arabidopsis thaliana*, together with *ABI1* inhibits *SnRK2.4* activity and regulates the growth of roots, thereby regulating the salt tolerance of plants (Krzywińska et al., 2016b). *OsPP108* belongs to subfamily A of rice, transgenic *Arabidopsis thaliana* with overexpression of *OsPP108* is insensitive to ABA and has strong resistance to salt stress and drought stress (Singh et al., 2015). *TaPP2C1*, a Group F2 Protein Phosphatase 2C Gene, confers resistance to salt stress in transgenic tobacco. Overexpression *TaPP2C1* transgenic tobacco showed ABA insensitivity (Hu et al., 2015). As can be seen, many members of the PP2C gene family have potential application prospect in plant stress resistance.

White birch (*Betula platyphylla Suk*) is one of the most important and precious broad-leaved trees. The wood of birch is hard and has a fine white texture, a wide range of uses in furniture, building materials and papermaking. However, birch is sensitive to salt stress. Results showed that the lethal salt concentration for birch is only 0.6%, among the lowest of 14 main afforestation species (Guifeng et al., 1998). Breeding new birch varieties with high salt tolerance has great significance to explore the suitable area for birch afforestation. It also helps to improve the ecological environment of arid and saline-alkali areas in western Northeast China.

In the current study, we identified and cloned a gene which had PP2C-type phosphatase domain from the birch transcriptome, and named it as BpPP2C1. Quantitative real-time PCR showed *BpPP2C1* had significantly response under salt stress and ABA treatment. The function of *BpPP2C1* gene was verified by both overexpression and knockout via transgenic experiments. During salt stress, overexpression lines showed obvious salt tolerance compared with the control, and knockout lines were sensitive to salt stress. The mechanism of salt tolerance associated with *BpPP2C1* is discussed.

## Materials and Methods

### Cloning and Bioinformatics Analysis of *BpPP2C1*

Total RNA was extracted from leaves of wild type *Betula platyphylla* according to manufacture protocol using RNA pure High-purity Total RNA Rapid Extraction Kit (BioTeke). cDNA synthesis was made using ReverTra Ace qPCR RT Master Mix with gDNA Remover (Toyobo, China). Protein coding sequence (CDS) of *BpPP2C* was obtained from reference genome of *Betula platyphylla*^1^. PCR primers P1 were designed for PCR cloning (All primer sequences used in this study were shown in Supplementary Table S1). PCR products were detected by 1% agarose gel electrophoresis and purified using TIAN gel Purification Kit (TIANGEN, Harbin, China) and sequenced (TSINGKE Biological Technology, Harbin, China). Physicochemical properties of amino acids of *BpPP2C1* were analyzed using ExPASy^2^. Protein secondary structure was predicted using PredictProtein^3^. Based on annotation of birch genome, 34 *PP2C* family genes including *BpPP2C1* were found. 80 *PP2C* family genes of *Arabidopsis* were found in TAIR^4^. The above *PP2C* family gene sequences are compared by ClustalX and infer phylogenetic trees by MEGA5.2.

### Subcellular Localization of *BpPP2C1* Protein

The CDS sequence of *BpPP2C1* gene was amplified with primer P2, and the PCR product fragment was digested and fused to the expression vector pCAMBIA1300. The onion epidermis was bombarded by a Gene Gun System PDS-1000/He and then cultured in the dark for 1 day to observe the fluorescent markers of GFP with a Zeiss Axio Imager A2 LED upright microscope.

### Expression Analysis of *BpPP2C1*

The birch seeds were sowed and cultured in growing pots (25 ± 3^L^). When the seedling height was about 10 cm, the stem tip, first leaf, second leaf, third leaf, fourth leaf, the upper part of the stem, lower part of the stem and the root were harvested. Each tissue was mixed with 5 seedlings, and 3 biological replications were set up for the analysis of tissue expression. To study the response of *BpPP2C1* to stress, 200 mM NaCI solution and 20% Polyethylene glycol (PEG) solution were used to irrigate the roots of birch seedlings, the root and the above ground are taken separately. To check for NaCl stress induction in the roots, we detected the expression levels of two SOD genes and three POD genes of roots after NaCI stress, and their expression levels showed varying degrees of up-regulation (Supplementary Figure S2). As for ABA treatment, plant leaves were sprayed with 100 mM concentration of ABA solution. The control group was sprayed with water. The time points were 0, 3, 6, 12, 24, 36, 48 h for all three treatments.

The total RNA of above tissues was extracted and reverse transcribed into cDNA as a template for qRT-PCR. Using primer 18S as internal reference gene, primer PP2C1-qPCR was used to amplify the *BpPP2C1*. qRT-PCR analysis was performed with PCR reaction system (SYBR^®^ Green Realtime PCR Master Mix - Plus-, Toyobo, volume 20 μl) and reaction program (95°30 s; 95°15 s 60°1 min 40 cycles; 95°30 s) running on the ABI7500 Real-time PCR (Thermo Fisher). Three technical repetitions were made for each reaction. The qRT-PCR results were analyzed by the 2^–ΔΔCt^ method.

### Construction of Overexpression Vector and Knockout Vector

The construction of overexpression vector was designed with primers P3 containing adapter. The full-length CDS sequence of *BpPP2C1* was amplified by PCR and ligated into 35S:PP2C1 overexpression vector by homologous cloning (Catalog. No. VK011-05, Beijing Viewsolid Biotech, China). A CRISPR Gene Knockout Kits based on SaCas9/gRNA system was used to construct *BpPP2C1* gene knockout vector (Catalog. No. vk005-101, Beijing Viewsolid Biotech, China). In the first exon of *BpPP2C1*, we determine the target-sgRNA next to protospacer adjacent motif (PAM). The primers P4 were designed at both ends of the target site to clone this fragment in wild-type Betula platyphylla. Sequencing confirmed that there was no single nucleotide polymorphism (SNP). Based on result of enzyme digestion *in vitro*, knockout efficiency of the gRNA target was 98%, which is much higher than the standard (Catalog. No. vk012, Beijing Viewsolid Biotech, China). Subsequently, we synthesized primers P5 according to the target site, and oligo dimer was formed by annealing primer then inserted into knockout vector.

### Transgenic Plants

The genetic transformation of birch was performed according to the previously publications (Huixin et al., 2019; Wang et al., 2019a,b). Seeds were collected from open pollination trees in seed orchard of *Betula platyphylla* in Northeast Forestry University. The seeds were dried, and sealed in plastic bags and stored in −20°C refrigerator. The wild type birch seeds were immersed in water for 2 or 3 days, and sterilized in 30% hydrogen peroxide for 15 min and rinsed with sterile water, before seeds were prepared to be used as the transgenic explants. *Agrobacterium tumefaciens* containing the 35s:*BpPP2C1* and *BpPP2C1*- knockout vector was used to infect the longitudinally cut seeds. Eventually, the explants were placed onto co-cultivation medium (WPM with 0.8 mg/L 6-BA, 0.02 mg/L NAA) under dark for 2 days and then planted on selective medium (WPM with 2.0 mg/L 6-BA, 0.02 mg/L NAA, 5.5mg/L hygromycin or glufosinate ammonium and 200 mg/L cefotaxime). After 30 days, resistant adventitious buds were induced (WPM with 2.0mg/L 6-BA, 0.02 mg/L NAA, 0.5mg/L GA,5.5mg/L hygromycin or glufosinate ammonium and 200 mg/L cefotaxime) and rooted (WPM + 0.4mg/L IBA) (Liu et al., 2019). After raised to 5 cm high in growth chamber, transgenic plants were transferred to the greenhouse. In May 2017, the seedlings were transplanted into the seedling pot for growth. At the beginning of May 2018, 30 plants with the similar growth trend were selected from each line of wild type (WT), overexpression (OE), and knockout (KO) and planted in a 21 × 21 cm growth pot. The substrate was peat soil, river sand and black soil (V/V) with ratio of 4:2:2. Transgenic plants were placed under the plastic greenhouse for routine management.

### Identification of Transgenic Plants

Since the genetic background of the seed used for transgenic is a half-sib family, we set up three wild type lines to get more accurate traits. In order to test whether the T-DNA is integrated in the plant genome, total DNA was extracted (DNAquick Plant System, TIANGEN) from leaves of 2-year-old transgenic *Betula platyphylla*. The primers P6 were designed from the resistance genes BAR contained in the T-DNA of over expressing vector, the primers P7 were designed from the resistance genes HPT for knock out vector. The PCR product was detected using 1% agarose gel. RNA was extracted from overexpression plants and WT plants for reverse transcription, and gene expression of *BpPP2C1* was detected by qRT-PCR. For *BpPP2C1* knockout plants, the primer P8 were used to amplify the target site and its adjacent sequences from transgenic plant DNA, PCR products were sequenced and the sequencing results were compared with wild-type by SeqMan^5^.

### Measurement of Physiological Traits of Transgenic Lines Under Salt Stress

Two years old *BpPP2C1* OE, KO and WT birch were subjected to salt stress treatment. There were 24 plants in each line. Half of them, 12 plants were irrigated with 0.4% NaCI solution for 12 days, and 12 plants were treated with water as controls. The soil was maintained at ion concentration 30 and 130 mol/L for control and NaCI treatment, respectively. The chlorophyll fluorescence kinetic parameters were measured by PAM-2500 chlorophyll fluorometers (WALZ, Germany). The sixth leaf counted from top of shoot was taken for measurement and were kept in dark for 20 min. Five biological replications were used. Salt damage index (d) was calculated as (d) = Σ (salt damage series × number of plants with corresponding salt damage level)/(highest salt damage level × total number of plants tested) (Zhao et al., 2017). Severity of salt damage: grade 0: no symptoms of salt damage; grade 1: mild salt damage, with a few leaf blade, Leaf tips, leaf margins or leaf veins turn yellow; grade 2: moderate salt damage, with 1/2 scorched leaf blade, leaf tip and edge; grade 3: severe salt injury, most of the leaf blade, leaf tip, leaf edge and vein turn yellow; grade 4: extremely severe salt damage, the leaves are scorched, and fall, plant death. The leaves were collected and freezed in liquid nitrogen for transcriptome and physiological indexes. Superoxide dismutase (SOD) activity was measured by SOD test kit (Nanjing Jiancheng Bioengineering Institute, A001-1), peroxidase (POD) activity was measured by peroxidase POD assay kit (Nanjing Jiancheng Bioengineering Institute, A084-3) and MDA content was measured by MDA test kit (Nanjing Jiancheng Bioengineering Institute, A003-1). After rehydration, plant height was recorded on July 4 and survival rate was investigated on October.

### Transcriptome Analysis

Nine leaf samples were collected from WT, OE, and KO under salt stress and each line had three biological replications. RNA was extracted from 27 samples by CTAB method, and the quality of RNA was detected by 1% agarose gel, NanoDrop 2000, and Agilent 2100 Bioanalyzer. Oligo (DT)—magnetic beads were used to enrich poly (a) mRNA. Fragment buffer was used to fragment the poly (a) mRNA. The fragments were reverse transcribed into cDNA library using test kit (Illumina, San Diego, United States). Illumina platform PE150 (Annoroad, China) was used for sequencing. TopHat was used to map clean reads against the reference birch genome^6^. The transcripts were assembled by Cufflink^7^. The expression matrix of fpkm value of each gene was obtained. Principal component analysis (PCA) was used to reduce the dimension of all samples, and the repeatability of each sample was evaluated, follow up analysis was conducted based on the three replications in each line. EdgeR was used to analyze the differentially expressed genes. log2 (fold change) ≥ 1 and FDR < 0.05 was up-regulated expression. log2 (fold change) ≤ −1 and FDR < 0.05 was down-regulated expression. Go function was enriched by Goatools^8^. When the corrected *P*-value (P_FDR) ≤ 0.05, it is considered that the go function has significant enrichment. Heatmap was produced by HemI_1.0.

## Results

### Bioinformatics Analysis of *BpPP2C1* Gene

**FIGURE 1:**
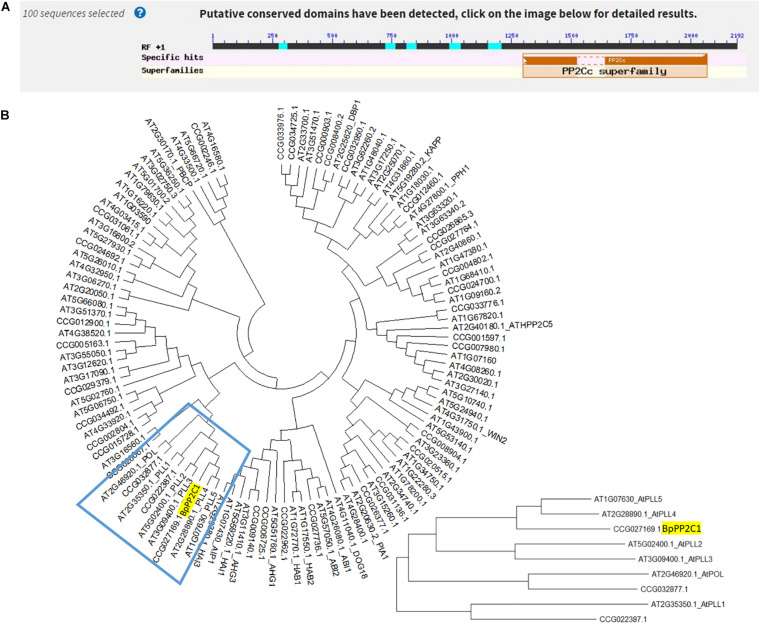
Bioinformatics analysis of *BpPP2C1*. **(A)** Sequence alignment of *BpPP2C1* using BlastX. **(B)** Phylogenetic tree of PP2C family genes in *Arabidopsis thaliana* and *Betula platyphylla*.

The CDS sequence of *CCG027169.1* gene of *Betula platyphylla* was compared in NCBI website. It was found that *CCG027169.1* gene contained a PP2C conserved domain and belonged to PP2C protein phosphatase family, then we named it *BpPP2C1* gene (Figure 1A). The *BpPP2C1* gene of *Betula platyphylla* is 2,190 bp in length, encodes 729 amino acids. The molecular weight of *BpPP2C1* is 81.13 kda; theoretical isoelectric point is 5.51; residual number of positive and negative charges is 84 and 105, respectively; molecular formula is C_3538_H_5510_N_1022_O_1121_S_26_; total number of atoms is 11,217; instability coefficient is 40.97; fat coefficient is 71.69; total average hydrophilicity is 0.564. *BpPP2C1* protein is composed of 20 kinds of amino acids, among them, serine (Ser) and leucine (Leu) have a higher content, 11.4 and 8.1%, respectively. Protein secondary structure prediction belongs to the mixed type protein, in which helix (H) accounted for 15.23%, fold (E) accounted for 13.44%, and ring (L) accounted for 71.33%. Besides, *BpPP2C1* protein contains 11 disulfide bonds, and each disulfide bond has a high prediction score, so it is predicted that the protein conformation is relatively stable. Thirty four genes were annotated as protein phosphatase 2C in *Betula platyphylla* in comparison with 79 PP2C family genes in *Arabidopsis thaliana*. It was found that PP2C family in *Betula platyphylla* and PP2C family in *Arabidopsis thaliana* had universal similarity (Figure 1B). The similarity of DNA sequence between *BpPP2C1* and *AtPLL5* gene was 64.7%, the highest in all candidate. Both and AtPOL, AtPol-like (PLL)1–4 genes converge into one branch which belong to PP2C subfamily C.

### *BpPP2C1* Protein Was Localized in Both Nucleus and Cell Membrane

**FIGURE 2:**
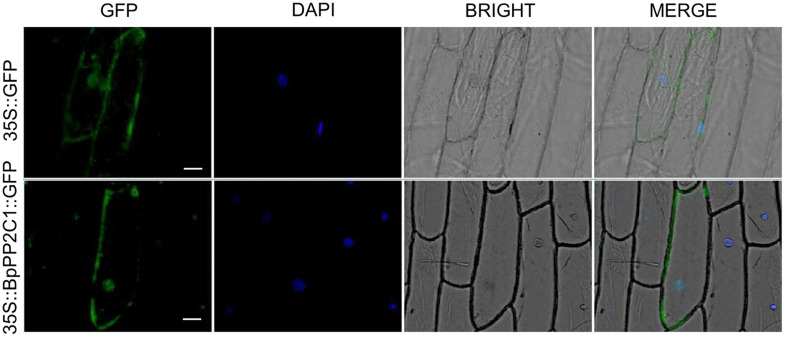
BpPP2C1-GFP was located in cell membrane and nucleus by analyzing for subcellular localization in onion epidermal cells. The scale is 50 μm.

The fluorescence signal of 35S:BpPP2C1-GFP appeared in both cell membrane and nucleus (Figure 2), while the 35S:GFP appeared in the cytoplasm, nucleus and cell membrane. This indicates that the *BpPP2C1* protein is localized to the membrane and nucleus of plant cell.

### Expression of *BpPP2C*1 Gene

**FIGURE 3:**
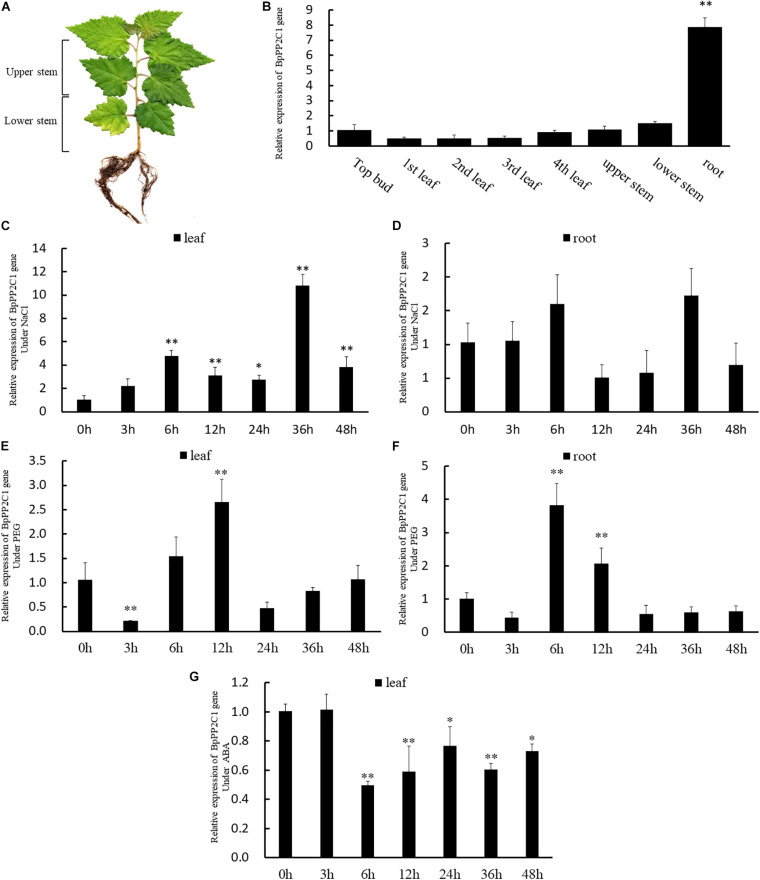
**(A)** Schematic drawing of the wild birch from 2 months old, stem is truncated from the middle divided into upper and lower stem. **(B)** *BpPP2C1* gene expression in different parts of leaf, stem, and root based on 2^–△△t^ method. Under NaCI stress, *BpPP2C1* gene expression in leaves **(C)**, and root **(D)** at different stress time points. *BpPP2C* gene expression in leaves **(E)**, and root **(F)** at different stress time points under Polyethylene glycol (PEG) stress. **(G)** *BpPP2C1* gene expression under ABA treatment at different time points. Asterisks indicate significant differences between transgenic lines and WT1 (Duncan multiple comparison method, *P < 0.05, **P < 0.01).

The expression of *BpPP2C1* gene in roots, stems and leaves of 2 month-old wild-type *Betula platyphylla* (Figure 3A) was widely expressed, but the expression levels were significantly different in different organs (Figure 3B). Taking the expression of apical bud as a control, the expression in roots highly significantly increased, which is more than 7 times that of apical buds, while the expression level in leaves and stems did not change significantly.

We also investigated the response process of *BpPP2C1* under salt and drought stress. Under NaCI stress, *BpPP2C1* gene expression in roots was still much higher than that of leaves (data not shown), but highly up-regulated in leaves. At time point 36 h, the expression level in the leaves was 10 times higher than that of the control group (Figure 3C). At the same time, the expression trend of roots was consistent with that in the leaves, reaching the highest at 36 h, 1.6 times higher than that of control roots (Figure 3D). Moreover, *BpPP2C1* gene was up-regulated under PEG stress, in roots, the expression level reached the highest at 6 h, which was 3.8 times than that of control (Figure 3F); in leaves, the expression level reached the highest at 12 h, which was 2.5 times higher than that of the control (Figure 3E). Under ABA hormones treatment, we found that *BpPP2C1* decreased significantly at 6 h, then recovered to a certain level in comparison with control (Figure 3G). *BpPP2C1* gene had the highest response to NaCI stress for wild type birch.

### Molecular Identification of Transgenic Plants

To study the function of the *BpPP2C1*, we constructed an overexpression vector and a knockout vector of the *BpPP2C1*, Agrobacterium-mediated method was used to transform the vector into wild-type birch zygotic embryos. DNA and RNA were extracted from leaves of 2-year-old transgenic *BpPP2C1* overexpression line (OE), *BpPP2C1* knockout line (KO) and control (WT). The phosphinothricin resistance gene (BAR) in the overexpression vector T-DNA was amplified by PCR (Supplementary Figure S1A). The results showed that all three OE lines had amplified bands, while three WT lines were had no band. It indicated that the T-DNA successfully integrated into the white birch genome. RT-qPCR showed that *BpPP2C1* gene expression in the overexpression line OE4, OE9 and OE10 was 77, 223, and 271 times higher than that of the WT, respectively (Supplementary Figure S1B). For KO lines, the PCR results of the co-transformed hygromycin resistance gene HPT showed that the T-DNA successfully integrated into the birch genome (Supplementary Figure S1C). In addition, the knockout target site and surrounding sequences were amplified by PCR, and PCR products were sequenced. The results showed that the sequence of the target site of the knockout lines did change. Compare to the original sequence, KO4 had an additional base A at the fourth base of the target site, making mutant *BpPP2C1* gene encodes 142 amino acids instead 729 amino acids. KO15 had multiple A at the fourth base followed by overlapping peaks in the sequencing signal. KO18 had an overlapping peak after the fourth base in target site. The overlapping peaks showed that there was multiple coding mode of nucleic acid sequence at the target site, so that the original protein encoded was reduced. The target site sequence of WT1, WT2, and WT3 did not change (Supplementary Figure S1D).

### Overexpression and Knockout of *BpPP2C1* Under Stress Treatment

#### Phenotypic Response Under NaCI Stress

**FIGURE 4:**
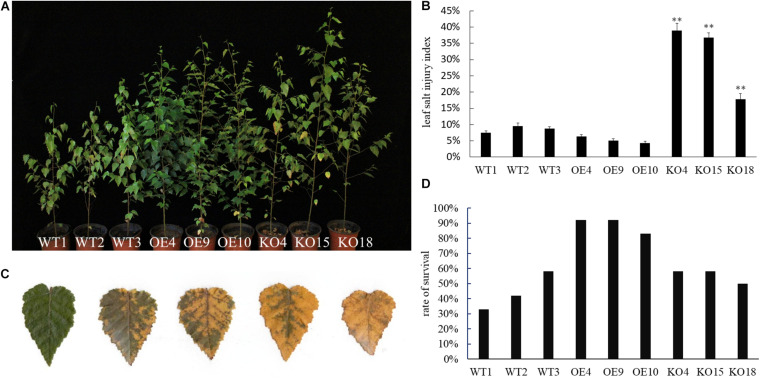
Phenotype observation of wild-type and transgenic birch after NaCI stress. **(A)** Phenotypic observation on transgenic and WT lines during salt stress treatment. **(B)** The leaf salt injury index of transgenic and WT lines after salt stress treatment. The error bars represent the standard deviation, asterisks indicate significant differences between transgenic lines and WT1 (*n* = 12, Duncan multiple comparison method, **P < 0.01). **(C)** Salt damage grade standard: Grade 0, leaves are healthy and undamaged; Grade 1, the edges of the leaves begin to turn yellow or wilting (the damaged area is less than 20% of the total leaf area); Grade 2, 50% of the total leaf area appears Yellow and wilting symptoms; level 3, yellowing and wilting symptoms appear in more than 80% of the total leaf area; level 4, leaf death. **(D)** Survey survival rate of wild-type and transgenic birch (*n* = 12) lines.

When plants were in the vigorous growth period in June, the 0.4% NaCI solution was applied to each line. At day 7, the KO line first developed salt damage symptom with a few leaf edges at the bottom of plant began to turn yellow. As the salt stress continued, the damage of the blade gradually spread from the leaf base to the entire blade, finally the leaves withered and dropped off; the WT lines was better than that of the KO strain, and its salt damage symptoms appeared 2 days later than that of the KO lines. At day 12, about 1/3 of the lower leaves of the KO lines dropped, and the leaves below about 1/2 height turned yellow. About 1/4 of the leaves of the WT lines were shed, while the overexpression lines only had yellow and fallen leaves near the base of the plant (Figure 4A). Comparing the salt damage index of each strain, it was found that the salt damage index of the overexpression lines was the lowest after the control, while the salt damage index of the knockout strain was the highest than that of other lines (Figures 4B,C).

Birch stressed by NaCI were rehydrated in water. Plant height growth and the relative height growth before and after stress were compared (Table 1). Among the WT, OE, and KO lines, the OE lines has the highest high growth after rehydration, especially OE9 and OE10 are significantly higher than the WT and KO lines (*P* < 0.05). The average height growth of the OE lines was 21.2 and 79.5% higher than that of the WT and KO lines, respectively. The height growth of KO lines was the lowest. KO15 and KO18 were significantly lower than WT and OE (*P* < 0.001). As for relative seedling height growth, the OE lines were also significantly higher than the WT and KO lines (*P* < 0.05). The average relative height growth of OE was 24 and 79% higher than those of WT and KO, respectively.

**TABLE 1:** Plant height growth of OEX, KOX, and NT lines during salt stress.

Line	Seedling height before and after salt stress (cm)		Height growth (cm)	Relative height growth
	0 day	25 days		
WT1	67.08 ± 7.05c	86.33 ± 7.71e	19.25 ± 1.23de	0.289 ± 0.028b
WT2	79.58 ± 3.73a	98.58 ± 6.58b	19.00 ± 3.32e	0.238 ± 0.034c
WT3	74.83 ± 6.34b	96.08 ± 3.73ab	21.25 ± 1.36cd	0.285 ± 0.023b
OE4	66.33 ± 4.05c	88.42 ± 4.07de	22.08 ± 1.61bc	0.334 ± 0.035a
OE9	81.08 ± 4.19a	107.17 ± 6.96a	26.08 ± 3.66a	0.321 ± 0.038a
OE10	68.33 ± 4.61c	92.25 ± 5.52cd	23.92 ± 3.07b	0.352 ± 0.051a
KO4	81.42 ± 5.62a	97.92 ± 6.44b	16.5 ± 2.14f	0.203 ± 0.028d
KO15	68.08 ± 4.87c	79.25 ± 3.29f	11.17 ± 1.86g	0.167 ± 0.038e
KO18	64.83 ± 2.83c	77.33 ± 3.54f	12.5 ± 2.25g	0.193 ± 0.036de

*HG (cm) = plant height at 25 days (cm)-plant height at 0 days (cm); Relative HG = HG (cm)/plant height 0 day (cm); Data indicate means ± STDEV (n = 12, P < 0.05, Duncan multiple comparison method).*

The survival rate of plants after re-watering revealed that the OE lines were higher than that of WT and KO (Figure 4D). Results indicated that under the stress of NaCI, OE showed strong resistance to salt compared with WT, while KO showed sensitivity. This suggested that *BpPP2C1* confers the salt tolerance of white birch.

#### Chlorophyll Fluorescence Parameters

**FIGURE 5:**
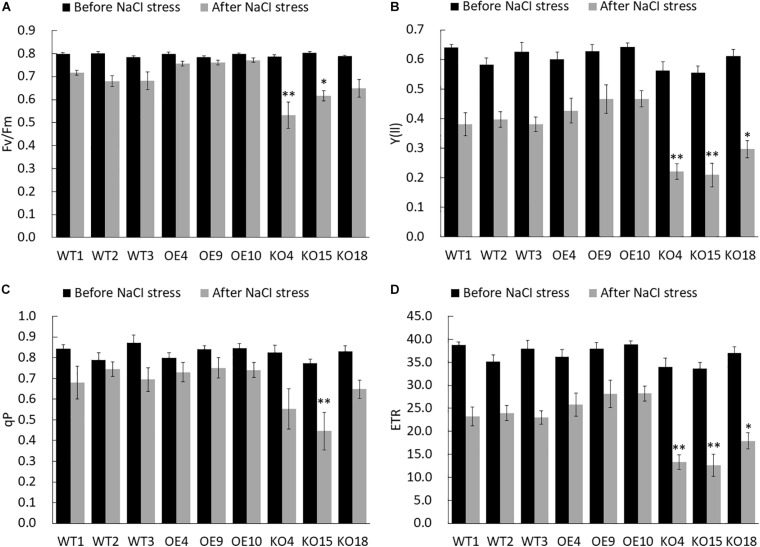
Chlorophyll fluorescence dynamic parameters of wild-type and transgenic birch before and after stress. **(A)** Fv/Fm (the maximum photochemical efficiency of PSII). **(B)** Y(ll) (effective photochemical quantum yield of photosystem II). **(C)** qP (Coefficient of photochemical fluorescence quenching). **(D)** ETR (electron transfer rate). The error bars represent the standard deviation, asterisks indicate significant differences between transgenic lines and WT1 (*n* = 12, Duncan multiple comparison method, *P < 0.05, **P < 0.01).

The chlorophyll fluorescence parameter is regarded as an internal probe to study the relationship between plant photosynthesis and environment. Here, PAM-2500 (WALZ, Germany) chlorophyll fluorometer was used to measure Fv/Fm (Maximum photochemical quantum yield of PS II), Y(ll) (effective photochemical quantum yield of photosystem II), qP (Coefficient of photochemical fluorescence quenching), and ETR (Electron transport rate) of each line before and after NaCI stress to analyze the salt tolerance of the transgenic lines. Fv/Fm reflects the potential maximum light energy conversion efficiency, which is the most important parameter of chlorophyll fluorescence. Under non-stress conditions, the change of Fv/Fm was very small, constant at about 0.8, while under stress conditions, this parameter decreases significantly (Figure 5A). However, comparing the Fv/Fm between different lines, the OE strain had the highest Fv/Fm, with an average value of 0.76, which was 10.1% higher than WT and 35.7% higher than KO, respectively.

The Y(II) value estimates the photochemical use of excitation energy in the light. There was no significant difference in the Y(II) value of each line in the unstressed control group, but after salt treatment, the Y(II) of each line decreased (Figure 5B). However, OE was slightly higher than that of the WT, while the knockout strain had the largest decrease, and its Y(II) value was about 50% lower than that of the WT.

qP is coefficient of photochemical fluorescence quenching, which to some extent reflects the oxidation degree of Q_A_, the primary electron acceptor of PS II. The decrease of qP was the result of the PS II reaction center being shut down. In the control group there was no significant difference in the qP value of each line. Under stress, the qP value of the OE lines was higher than others, and the overall qP of the KO strain was lower than that of WT and OE (Figure 5C). The KO15 line reached extremely significant Level.

ETR reflects the efficiency of electron transfer. The ETR in the control group did not change significantly. Under stress, the average ETR of OE lines is 29.5 and 33.9% higher than WT and KO lines, respectively. KO was significantly lower than WT (Figure 5D).

The overexpressing *BpPP2C1* transgenic birch maintains better photosynthetic fluorescence characteristics under salt stress, while the birch with the knocked out was sensitive to salt. Chlorophyll fluorescence parameters were greatly reduced.

#### Physiological Indicators

Peroxidase (POD) catalyzes H_2_O_2_ to oxidize other substrates to produce H_2_O. It is an important enzyme to eliminate the damage of oxygen free radicals in plants, and associated with the resistance to salt stress of plants. There is no significant difference in the POD activity of control lines Supplementary Figure S3A. Under stress, the POD activity increased. The POD activity of OE is 41.9% larger than that of WT. OE4 and OE9 were the highest lines. POD activity of KO was 19.4 and 43.2% lower than WT and OE, respectively.

Superoxide dismutase (SOD) can reduce the damage of active oxygen or other peroxides to the cell membrane system by catalyzing the disproportionation reaction of superoxide anion free radicals, thereby effectively improving the anti-stress function of plants. There is no significant difference in the SOD activity for the control lines Supplementary Figure S3B. Under salt stress, the SOD activity of each line increased. but compared to WT and OE, the SOD activity of KO was the lowest, reduced by 28.2 and 35.8% in comparison with WT and OE.

**FIGURE 6:**
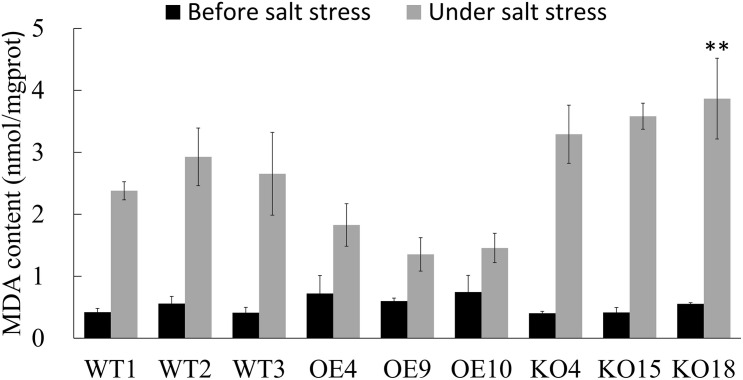
The content of Malondialdehyde (MDA) of wild type and transgenic *Betula platyphylla* before and after stress was measured. Asterisks indicate significant differences between transgenic lines and WT1 (Three independent biological repetitions, **P < 0.01, Duncan multiple comparison method).

Malondialdehyde (MDA) is the final decomposition product of membrane lipid peroxidation. MDA content reflects the degree of the damage suffered by adversity stress. After salt stress, the MDA content of each line increased greatly (Figure 6). As for all lines, MDA content of OE was the lowest, which was 44.4% lower than that of WT, and the content of KO was the highest, which was 33.3% higher than that of WT.

Under salt stress, OE plants had better SOD and POD vigor to resist the poison of oxidative stress and maintain the physiological activities of the plants. The activity of SOD and POD of *BpPP2C1* knocking out birch was worst, resulting in more serious membrane damage to the plant, indicated by the content of MDA.

### Transcriptome of Transgenic Betula Platyphylla

Leaves of WT, OE, KO strains after salt stress treatment were collected. The transcriptome of 27 samples was determined and analyzed. In total, 112.60 Gb clean data was obtained for each sample. The clean data reached 3.56 Gb, and the Q30 base was 93.35% and above. Align the clean reads of each sample with the birch reference genome, and the comparison efficiency ranges from 86.95 to 88.60%. Based on the comparison results, we conducted a repeatability analysis, and selected the best repeatability materials among the wild-type, over-expression lines, and knockout lines for subsequent analysis. We identified differentially expressed genes (DEGs) according to their expression levels in different samples, and perform functional annotation and GO enrichment analysis.

**FIGURE 7:**
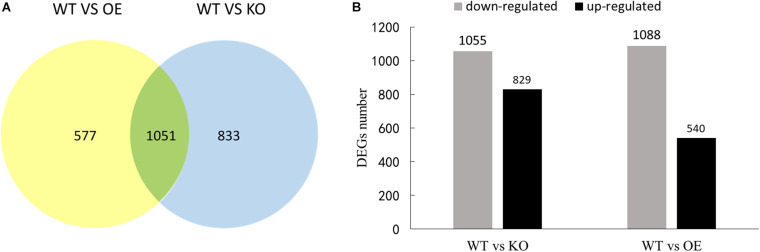
The number of differential genes in the transcriptome of the birch transgene. **(A)** Venn diagram of differentially expressed genes. **(B)** The number of differential genes up-regulated and down-regulated in transgenic lines compared to WT.

Compared with WT, 1628 and 1884 DEGs were found in OE and KO, respectively. Among identified DEGs, 1051 genes are shared by both OE and KO strains (Figure 7A). In the KO line, there were 829 up-regulated genes and 1,055 down-regulated genes, while 540 up-regulated genes and 1,088 down-regulated genes were found in the OE lines (Figure 7B).

**FIGURE 8:**
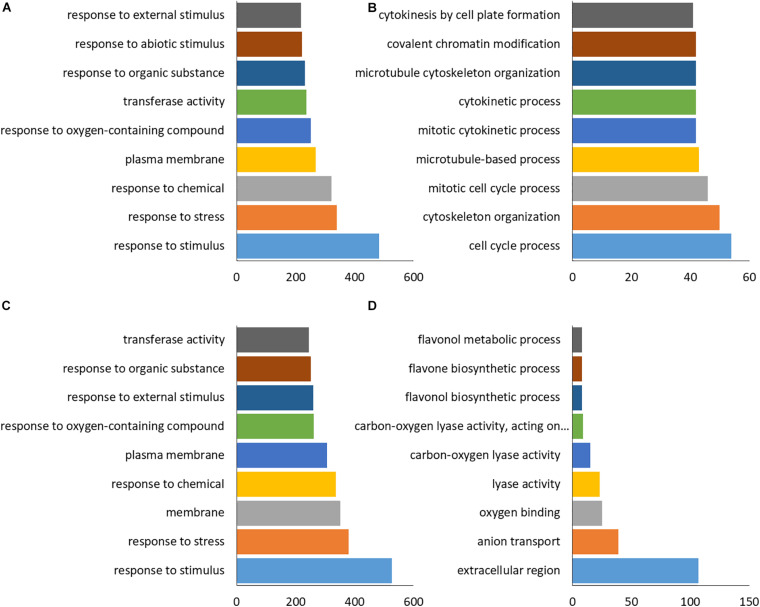
Differential gene GO enrichment of transgenic plants compared with wild-type plants. GO annotation of differential genes, selected by the pathways with fdr <0.05, The abscissa shows the number of genes in the corresponding pathway. **(A)** Down-regulated genes in KO. **(B)** Up-regulated genes in KO. **(C)** Down-regulated genes in OE. **(D)** Up-regulated genes in OE.

GO enrichment analysis of differential genes found that there were a large number of genes down-regulated by OE and KO are enriched in stress-related pathways, including response to external stimulus, response to stress, response to oxygen-containing compound, response to chemical (Figures 8A,C). It indicates that transgenic plants and wild type have different ways of coping with salt stress at the RNA level. In addition to stress-related pathways, extraordinary genes are also concentrated on plasma membrane and transferase pathway, it showed that the effect of *BpPP2C1* gene on plants is likely to be manifested in signal transduction. For the up-regulated genes, KO and OE showed different types of enrichment. A large number of genes up-regulated in KO were related to the cell cycle (Figure 8B), including the mitotic cell cycle process, cytoskeleton organization, microtubule-based process, cytokinesis by cell plate formation. In addition, covalent chromatin modification pathway was also enriched in KO. Unlike KO lines, genes up-regulated in OE were concentrated in the extracellular region, anion transport, oxygen binding, lyase activity, and the metabolism process of flavanol and flavone (Figure 8D).

**FIGURE 9:**
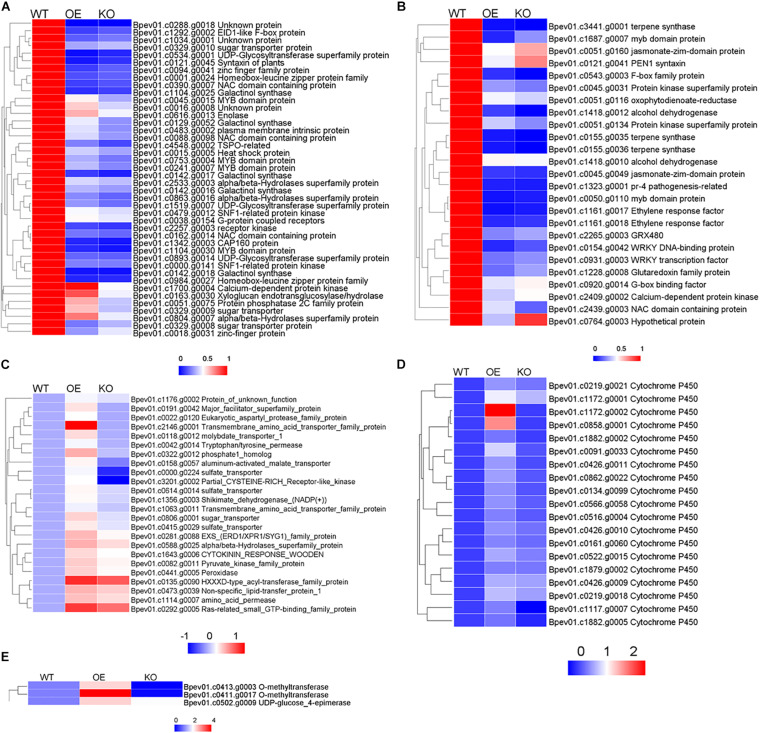
Thermogram analysis of salt stress related genes in differentially expressed genes. **(A)** Salt stress genes related to ABA pathway. **(B)** Salt stress genes related to JA, SA, ET pathways. **(C)** Salt stress genes related to anion transport. **(D)** Salt stress genes related to oxygen binding. **(E)** Salt stress genes related to flavanol synthesis. In each group, the average FPKM value of each member was taken, calculate the ratio of OE or KO compared with WT.

As for down-regulated DEGs related to salt stress in OE and KO, many genes were related to the anabolism and signal transduction of hormones such as abscisic acid (ABA), jasmonic acid (JA), salicylic acid (SA), and ethylene (ET) (Figures 9A,B). It shows that compared with the wild type, in the transgenic plants, hormones related to salt stress (ABA, JA, SA, ET), their metabolism and response were likely to undergo huge changes, resulting in the plants showing different degrees of salt tolerance. These hormone-related DEGs in the stress pathway were annotated, contained many stress-related transcription factors such as: MYB, NAC, WRKY, and zinc finger protein. Simultaneously, protein kinases, protein phosphatases, oxidoreductases, synthetases, alcoholases, transferases, and heat shock proteins were also identified.

In OE plants, there were significantly up-regulated gene compared with WT and KO plants enriched on anion transport, oxygen binding and flavanol synthesis pathways. Anion transport genes mainly included sulfate transporter, alpha/beta hydrolase and transmembrane protein were up-regulated (Figure 9C). Oxygen binding proteins included multiple cytochrome P450 family members, which play an important role in stress were up-regulated (Figure 9D). Genes related to flavanol metabolism included UDP glucose -4-epimerase, O-methyltransferase were up-regulated (Figure 9E). Up-regulation of these pathway genes may confer salt tolerance to overexpressing *BpPP2C1* plants.

## Discussion

### Expression of *BpPP2C1* Gene Has Tissue Distribution Specificity Under Salt Stress

Tissue distribution specificity of plant may be closely related to gene function. In 2-month-old wild-type birch, *BpPP2C1* is mainly expressed in mature stems and highly expressed in roots (Figure 3B). Roots is the first organ of plant to receive the salt stress signal, which then affects the growth of the above-ground parts (Fu et al., 2019). *BpPP2C1* is a phosphate signal transmitter, and its abundant expression in roots is likely to be a great significance for plants to respond to salt stress. As a regulatory factor in the MAPK cascade signal pathway, PP2C protein phosphatase may participate in the transduction and amplification of extracellular stimulus signals in root (Brock et al., 2010), cooperating with aerial parts to adapt to salt stress environment (Colcombet and Hirt, 2008). The expression trend of *BpPP2C1* gene in roots and leaves during the salt stress was very consistent, both gradually increased, leap up to 1.7 times in roots but up to 10 times in leaves (Figure 3C). It shows that during the process of environmental changes, the signal transduction of *BpPP2C1* be activated more in the leaves rather than in roots.

### Expression of *BpPP2C1* Gene Increases Salt Tolerance

*BpPP2C1* gene can highly express in response to salt stress (Figures 3C,D). Experiment of salt stress treatment was carried out on *BpPP2C1* overexpression lines, knockout lines and wild type of birch. From the most intuitive phenotypic traits such as salt damage index, relative/absolute high growth, and plant survival rate after stopped treatment, OE strain showed strong resistance to salt, while knockout plants KO showed salt-sensitive. Chlorophyll fluorescence parameters are generally used to characterize the intrinsic action of photosystem II (PSII), which is interrelated with the photosynthetic capacity (Hao et al., 2012). The absorption, transmission, dissipation and distribution of light energy in leaves during stress can be reflected in chlorophyll fluorescence parameters, indicating the damage degree of the plant (Zhang, 1999; Hao et al., 2012). Decreased Fv/Fm (Maximum photochemical quantum yield of PS II), Y(ll) (Effective photochemical quantum yield of photosystem II), qP (Coefficient of photochemical fluorescence quenching), and ETR (Electron transport rate) reflect that PSII activity decrease, electron transport is disrupted, photochemical efficiency is reduced and photosynthesis is inhibited (Maxwell and Johnson, 2000; Erhard et al., 2008). There was no significant difference between the transgenic line and the wild-type control before the stress, but after the stress, chlorophyll fluorescence parameters of the KO were lower than those of the WT, while those of OE are higher than WT and KO strain (Figure 5). It shows that the photosynthesis efficiency of OE strain was maintained under salt stress.

High concentration of salt damages the electron transport chains of different subcellular structures of plant cells, leading to the production of reactive oxygen species (ROS), damaging proteins, membrane lipids and other cellular components, and producing secondary oxidative stress (Moller, 2001; Bartolomé and Mercedes, 2013). The elimination of ROS in plants mainly relies on enzymatic reactions. Superoxide dismutase (SOD) can reduce the damage caused by ROS by catalyzing the disproportionation reaction of superoxide anion radicals (Apel and Hirt, 2004). Hydrogen peroxide (H_2_O_2_) is a cytotoxic substance produced in the redox reaction catalyzed by oxidase. Peroxidase (POD) catalyzes H_2_O_2_ to directly oxidize phenolic or amine compounds to eliminate the harmful effects of hydrogen peroxide (Meloni et al., 2003; Cantarello et al., 2005). In this study, we found that the OE lines under stress treatment had higher POD and SOD activities, and the KO lines had low POD and SOD activities than wild type (Supplementary Figure S3). MDA, a stable product of the membrane lipid peroxidation, can reflect the damage degree and the senescence process of plant (Rattan et al., 2020). It can be seen that after being subjected to salt stress, compared with the wild type, the transgenic birch with the *BpPP2C1* gene knocked out has a higher MDA content, and the overexpressing the *BpPP2C1* gene had a lower MDA value (Figure 6). These experimental results showed that the expression of the *BpPP2C1* gene improves the antioxidant defense system, thereby protecting transgenic plants from salt stress by reducing damage caused by oxidative stress.

### BpPP2C1 Gene May Play a Role in Salt Stress Though ABA Signaling Pathway

Previous studies have found that the PP2C family number are widely involved in abiotic stress though ABA Signal pathway. Under salt stress, ABA is synthesized rapidly, and firstly binds to ABA receptor *PYR/PYL* family proteins, inhibits the activity of protein phosphatase PP2Cs, releases the activity of SnRK2 protein kinase (Park et al., 2009; Zhao et al., 2018). Then the transcription factor which interact with *SnRK2* protein kinase is phosphorylated and combined with the response element AREB of the downstream gene promoter, and finally the ABA response gene is activated and expressed (Hubbard et al., 2010; Krzywińska et al., 2016a). It is a well-known that members of *Arabidopsis* PP2C family A subfamily group play an important negative regulatory role in ABA signal transduction (Nishimura et al., 2010). In contrast, the *OsPP108* gene is a gene of the PP2C family A subfamily in rice. Transformation of the *OsPP10*8 gene into *Arabidopsis* shows that the *OsPP108* gene is negatively regulated in the ABA signaling pathway, but positively regulated in salt stress and drought stress (Singh et al., 2015). *TaPP2C1* is a member of the F subfamily of the tobacco PP2C family. The overexpression of *TaPP2C1* transgenic tobacco shows ABA insensitivity and significant salt resistance (Hu et al., 2015). *AtPP2CG1* belongs to Arabidopsis thaliana protein phosphatase 2C G Group 1. The expression levels of AtPP2CG1 in the ABA synthesis-deficient mutant abi2-3 were much lower than that in WT plants under salt stress suggesting that the expression of *AtPP2CG1* acts in an ABA-dependent manner. Over-expression of *AtPP2CG1* led to enhanced salt tolerance, whereas its loss of function caused decreased salt tolerance. These results indicate that *AtPP2CG1* positively regulates salt stress in an ABA-dependent manner (Liu et al., 2012). The overexpression of the *MsPP2C* gene isolated from alfalfa Zhongmu conferred salt-tolerance in Arabidopsis, Fluorescence quantitative PCR showed that salt stress, abscisic acid (ABA), and drought stress induced the expression of *MsPP2C* (Guo et al., 2018). It can be found that PP2C, a huge gene family, encodes functional proteins with different physiological characteristics. The role of many PP2Cs is still unknown, especially for the PP2Cs C subfamily. Previous reports on the PP2Cs C subfamily mostly regulate the phenotype of plants: It was found that both AtPOL and AtPLL1 regulate cell differentiation, and the expression of genes has a significant effect on the phenotype; AtPLL4 and AtPLL5 appear to regulate leaf development (Song and Clark, 2005). Here, we found a PP2C subfamily C gene in birch named *BpPP2C1* may involve in ABA signal transduction and abiotic stress. The expression of *BpPP2C1* was down-regulated after ABA treatment in wild-type birch (Figure 3G). Transcriptome analysis of *BpPP2C1* overexpression lines and knockout lines found that a large number of salt stress-related genes were down-regulated (Figures 8A,C). Among them, many genes involved in the ABA signaling pathway are included: transcription factors such as MYB transcription factor family, zinc finger protein, NAC family, and homeodomain-leucine zipper protein (HD-Zip) (Phang et al., 2008; Agarwal and Jha, 2010). It also contains heat shock proteins and protein kinases annotated in the ABA pathway which included SNF1-related protein kinases, a key regulator of the plant response to osmotic stress (Krzywińska et al., 2016a). It can be seen that the expression of *Betula platyphylla BpPP2C1* has an impact on the ABA signal transduction pathway under salt stress, disrupting the expression of ABA signal transmission and response related genes. This system may be working through the transmission of phosphate signals, activating protein kinases to modify the activity of transcription factors, then the expression of downstream resistance-related genes is activated. Eventually changes the salt tolerance of PP2C1 gene overexpression lines and gene knockout lines.

### The Salt Tolerance of OE Strains May Be Related to the Up-Regulated Expression of Oxidative Stress-Related Genes

Compared with the wild type, the up-regulated genes of KO and OE strains showed different enrichment types. In the salt-tolerant OE lines, genes enriched in extracellular region, anion transport, oxygen binding, lyase activity, and flavanol biosynthetic process were significantly up-regulated (Figure 8D). Genes related to anion transport like peroxidase, sulfate transporter, sugar transporter, molybdate transporter, aluminum-activated malate transporter, transmembrane amino acid transporter were up-regulated in OE strains (Figure 9C). In addition, it is worth noting that genes code cytochrome enzyme P450 family protein were significantly up-regulated (Figure 9D; Mao et al., 2013). This gene family involved in salt tolerance, they catalyze various oxidative reactions and are of great significance to plant metabolism (Brankova et al., 2007; Mizutani, 2012; Weifang et al., 2017). In addition to the enzymatic reactions involved in POD and SOD, which can alleviate the oxidative damage caused by salt stress, flavonoids are a major class of non-enzymatic active oxygen scavengers, and their synthesis ability is significantly enhanced under salt stress (Tattini et al., 2004; Chen et al., 2018; Sheng et al., 2020). Among them, flavanols are resistant to the most oxidizing (Apel and Hirt, 2004; Treutter, 2006). Transcriptome analysis showed that the expression of flavanol biosynthetic genes were up-regulated (Figures 8D, 9E). The OE line may enhance the ability to remove active oxygen by increasing the content of flavanols, thereby improving the salt tolerance of plants. Within the up-regulated oxidative stress-related genes, there are nine genes that show opposite expression patterns between OX and KO. It is possible those genes related to PP2C1 (Supplementary Figure S4). These genes were: major facilitator superfamily protein, sulfate transporter, aluminum-activated malate transporter, eukaryotic aspartyl protease family protein, partial CYSTEINE-RICH Receptor-like kinase, o-methyltransferase, cytochrome P450, which can be studied in subsequent experiments.

## Conclusion

In summary, the *BpPP2C1* gene encodes a protein phosphatase of the PP2C family, which is in the nucleus and cell membrane, highly expressed in roots and responds to ABA and salt stress. The overexpressing *BpPP2C1* gene are obviously salt-tolerant, while the strains that knock out the *BpPP2C1* gene are sensitive to salt. Transcriptome analysis showed that a large number of salt stress-related genes were differentially expressed in *BpPP2C1* transgenic lines, explaining that the reason for the difference in salt tolerance of transgenic plants may be related to the signal transmission in the ABA, JA, SA, ET pathways. Overexpression of *BpPP2C1* may confers white birch salt tolerance by reducing oxygen stress damage, referencing the up-regulated expression of gene related to metabolic activity of flavanols, anion transport, and oxidative stress.

## Data Availability Statement

The datasets presented in this study can be found in online repositories. The names of the repository/repositories and accession number(s) can be found below: NCBI Sequence Read Archive, accession no: PRJNA684481.

## Author Contributions

GL and JJ designed the research. BX conducted the experiments, data analysis, and wrote the manuscript. CG and TZ performed transcriptome data analysis. QZ, QY, GL, and JJ revised the manuscript. All authors read and approved the manuscript.

## Conflict of Interest

The authors declare that the research was conducted in the absence of any commercial or financial relationships that could be construed as a potential conflict of interest.

## Footnotes

1 https://genomevolution.org/CoGe/GenomeInfo.pl?gid=35079

2 http://web.expasy.org/protparam/

3 https://predictprotein.org/

4 https://www.arabidopsis.org

5 https://www.dnastar.com/

6 http://tophat.cbcb.umd.edu/

7 http://cufflinks.cbcb.umd.edu/

8 https://github.com/tanghaibao/GOatools
