# Supplementary material for: Functional Study of BpPP2C1 Revealed Its Role in Salt Stress in Betula platyphylla
Source: Front Plant Sci. 2021 Jan 14;11:617635. doi: 10.3389/fpls.2020.617635 (PMC7841333; doi:10.3389/fpls.2020.617635)
Supplement: Supplementary file 1 [file Data_Sheet_1.docx]

Supplementary Material

# Supplementary Figures


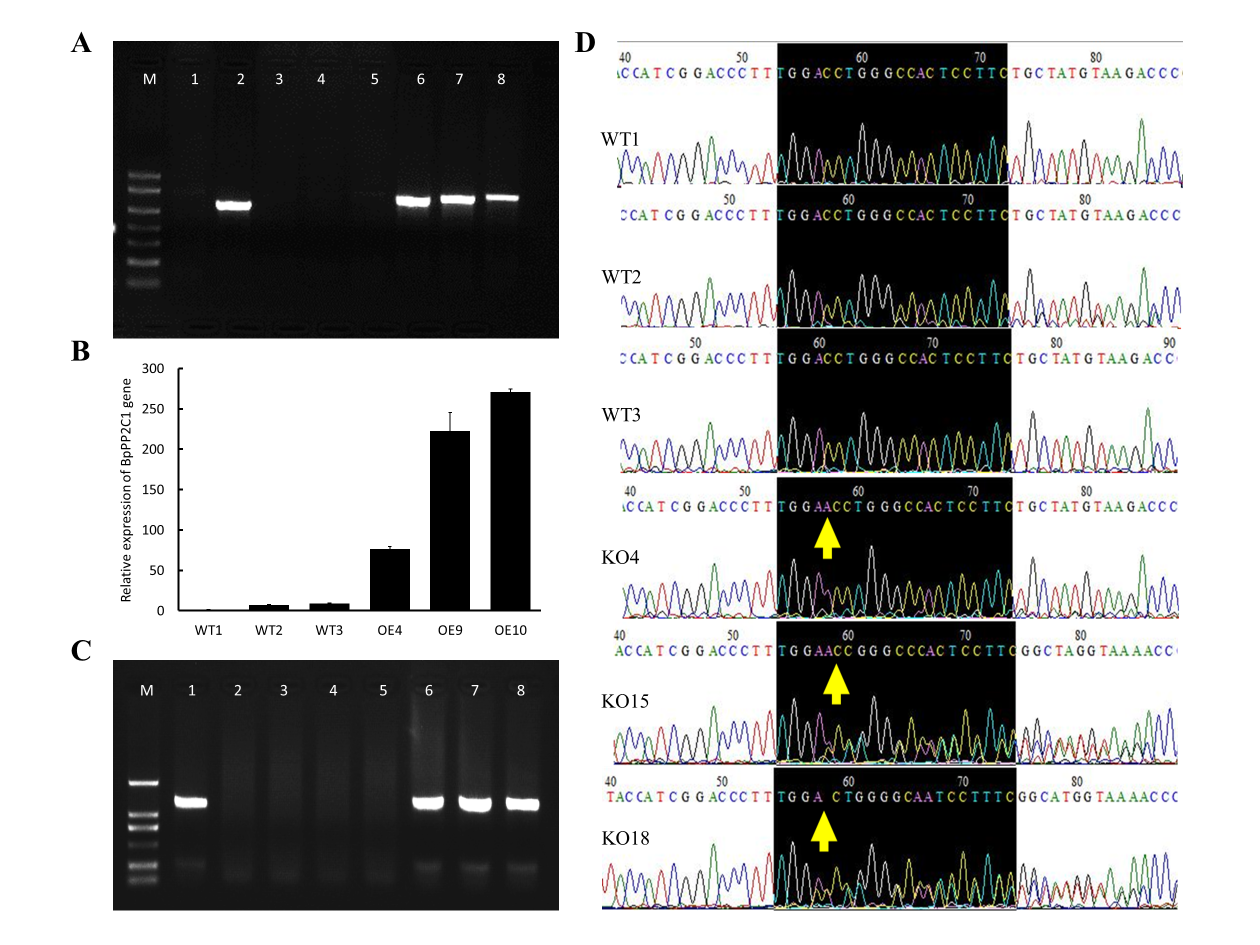


**Supplementary Figure S1.** (**A**) Amplification of resistance gene BAR (552bp) in overexpressed transgenic lines. M. DL2000 DNA ladder (2000,1000,750,500,250,100bp). 1. Water control 2. Positive plasmid control 3. WT1 4. WT2 5. WT3 6. OE4 7. OE9 8. OE10. (**B**) Expression of PP2C1 gene in wild-type and overexpression transgenic lines. (**C**) Amplification of resistance genes HPT in knockout transgenic lines. (**D**) Sequencing of the target site of knock out line, the black background indicated the knockout site, and the yellow arrow indicated the position where the target sequence began to change.


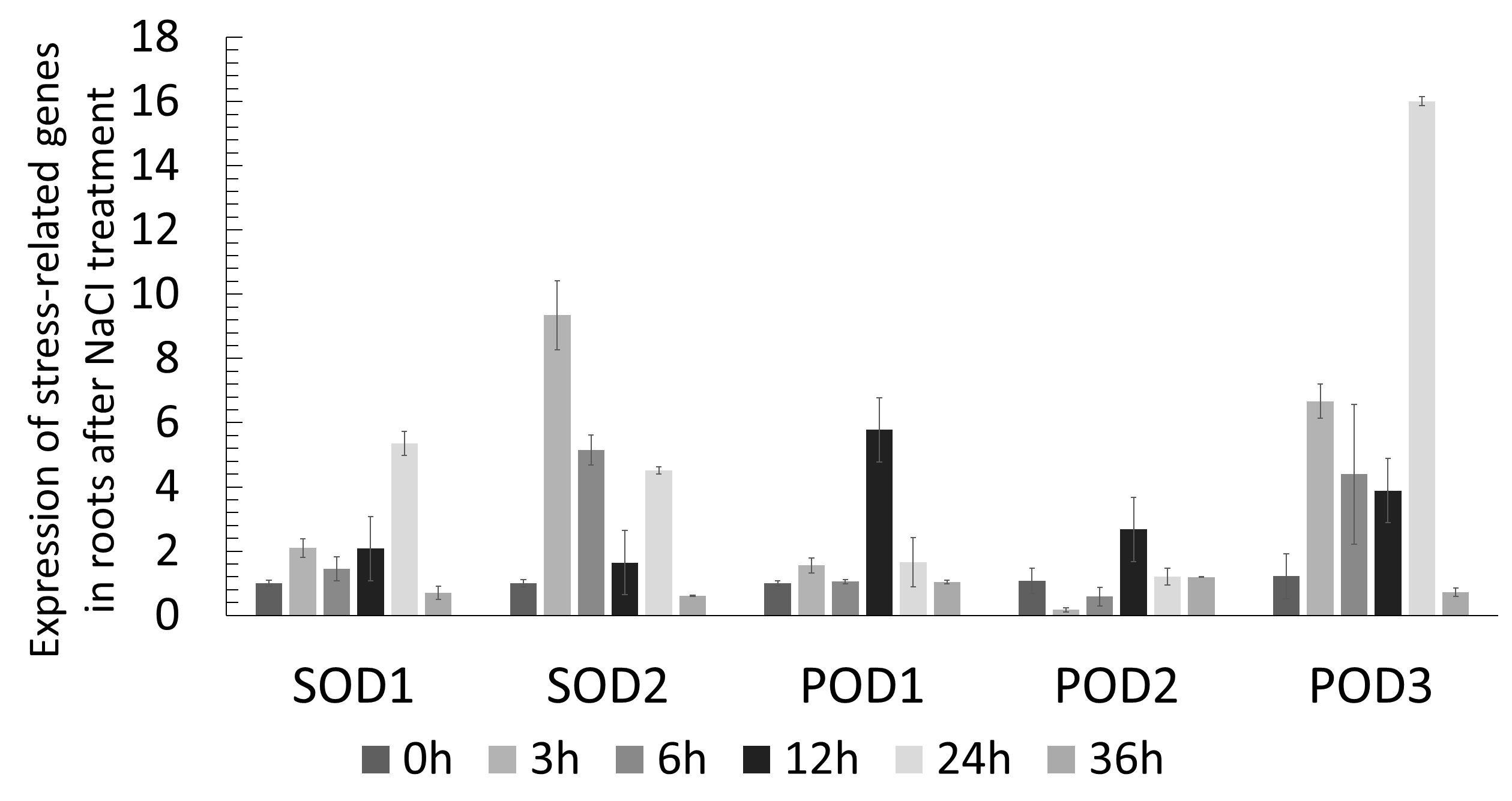


**Supplementary Figure S2.** Expression of stress-related genes in roots after NaCI treatment based on 2^-△△t^ method.


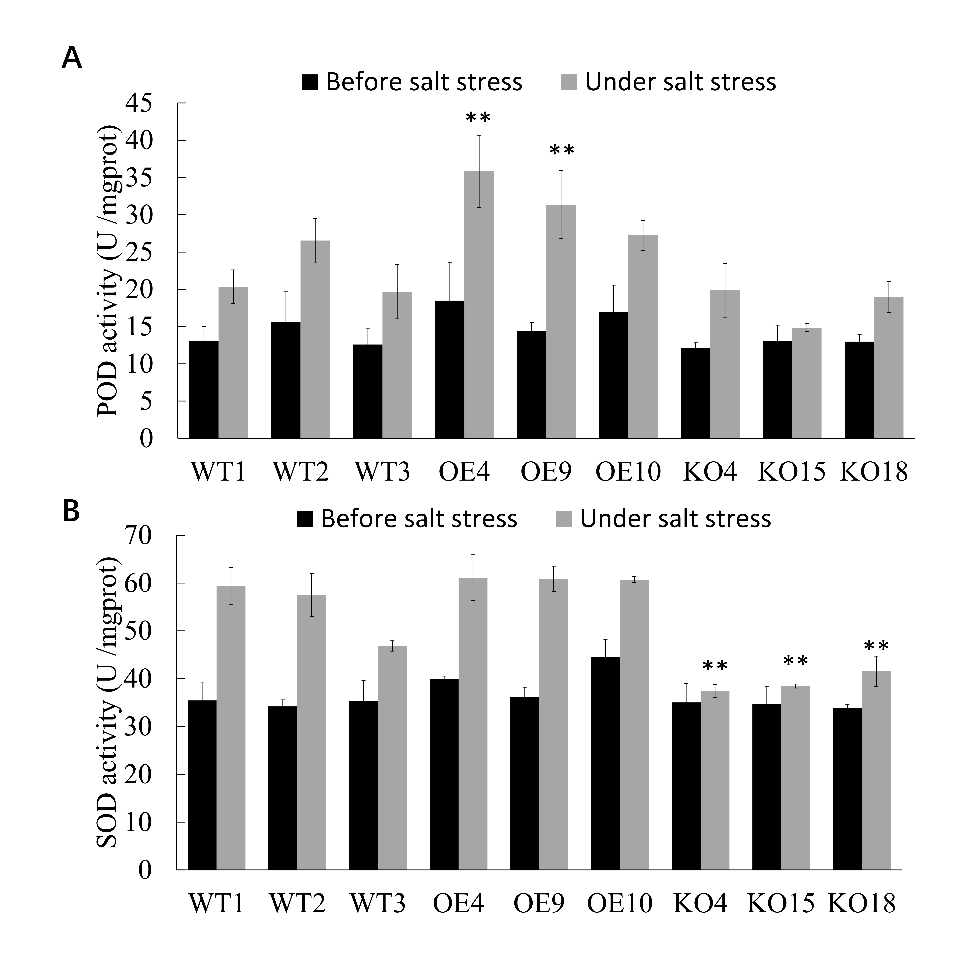


**Supplementary Figure S3.** Pysiological indexes of wild type and transgenic *Betula platyphylla* before and after stress. (A) The activity of superoxide dismutase (SOD) was measured. (B) The activity of Peroxidase (POD) was measured. (Three independent biological repetitions, asterisks indicate significant differences between transgenic lines and WT1. **P<0.01, Duncan multiple comparison method).


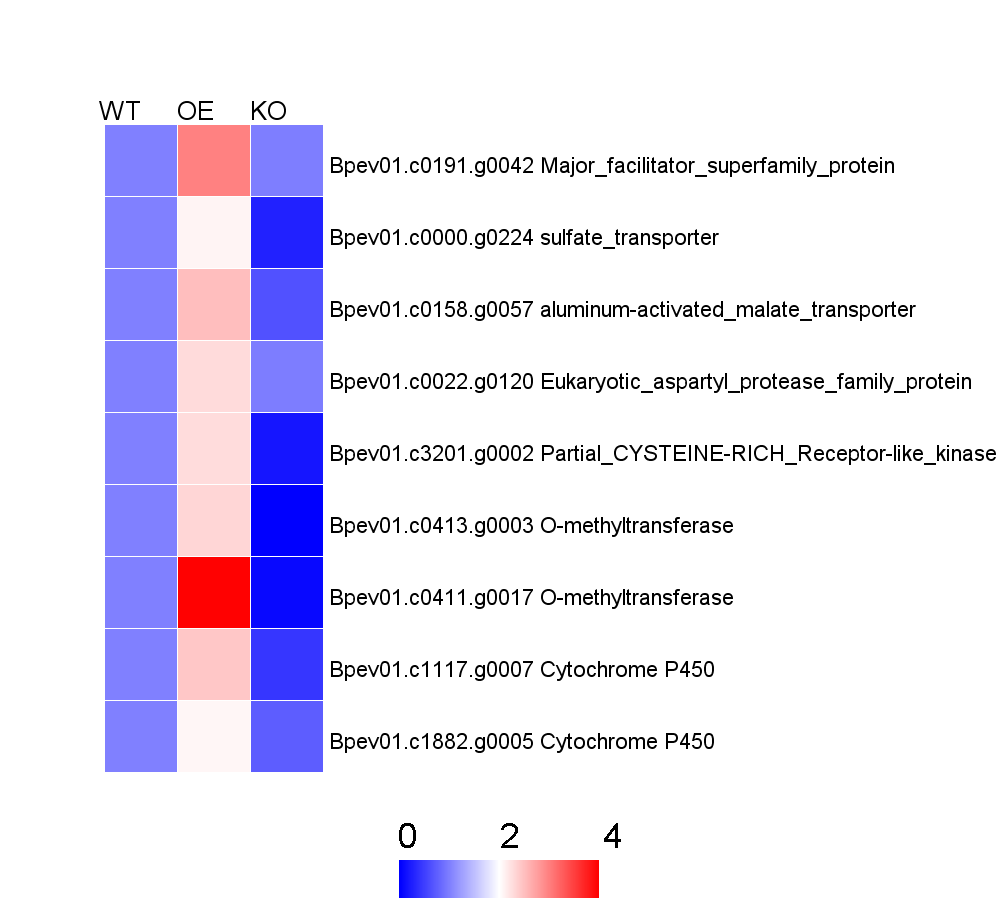


Supplementary Figure S4. The up-regulated oxidative stress-related Genes, showed opposite expression patterns between OE and KO, in each group, the average FPKM value of each member was taken, calculate the ratio of OE or KO compared with WT.

# Supplementary Table

Supplementary Table S1. The primers involved in this article

| Primer name | Forward primer | Reversed primer |
| --- | --- | --- |
| P1 | ATGGGTAATGGTTTTGGAAAGCTGA | TTATACACATGACCTCCATATCCTTCC |
| P2 | CGGGGTACCATGGGTAATGGTTTTGGAAAGC | CGCGGATCCTACACATGACCTCCATATCCTTC |
| 18S | GAGGTAGCTTCGGGCGCAACT | GCAGGTTAGCGAAATGCGATAC |
| PP2C1-qPCR | CTGCTAAAAGCTCAGCATTACCCC | CAACATGAACCAGCATTTACACCTTTAC |
| P3 | GCAAGTTCTTCACTGTTGATAATGGGTAATGGTTTTGGAAAGCTGA | TGATTTCAGCGTACCGAATTGTTATACACATGACCTCCATATCCTTCC |
| P4 | ATGGGTAATGGTTTTGGA | TGGTCAGAGTAACCCTTCTCG |
| P5 | TTGGAAGGAGTGGCCCAGGTCCA | AACTGGACCTGGGCCACTCCTTC |
| P6 | ATGAGCCCAGAACGACGC | TCAAATCTCGGTGACGGGC |
| P7 | CTATTCCTTTGCCCTCGGACG | AAGCCTGAACTCACCGCGA |
| P8 | ATGGGTAATGGTTTTGGA | TGGTCAGAGTAACCCTTCTCG |

# Supplementary Data

CDS sequence of BpPP2C1

ATGGGTAATGGTTTTGGAAAGCTGACAGTGTGCTTCACAGGCGGCGGAGAGGGCCGCCGGAGGAAGGACATACCAGCTTTACCATCGGACCCTTTGGACCTGGGCCACTCCTTCTGCTATGTAAGACCCGACCCGAGTCGCCTCTCGTCCTCCAAGGTCCACTCCGAAGAGGCCACCACCTTCAAAACTATCTCCGGTGCTTCGGTGAGCGCCAACACGTGGACACCTCTGTCCACAGACATCATAGACGTCTACTCCTACAACGGTATCGACCGAGCTGCAGCGTTCGAGAGCTCAACCTCCTTCGCTTCGCTTCCTCTGCAACCGATTCCGAGGAATTTGATGAATTCAGGTCCGATTTCCGGCAGCCTGTACGGAATTCCTGGCTCGGGTCCGTTGGAGAGAGGATTTCTATCGGGCCCGATTGAGCGGGGCTTCATGTCTGGCCCGTTGGATCGCGGGCTGTACTCGGGTCCGTTCGAGAAGGGTTACTCTGACCAGTTCCAGAGGAGCTTCTCCCATGGGGCTTTTGCGTTCAGGCCCAGATCGAGAAAAGGGAAGCTGATTCAGGTCCTCCAGAGAGCGATATCGAAGGCTATGTCTAGGGGGCACCAAAACTCGGTCGTGGCTCCGATTAAATCCGCCGTGGTAAAAGAACCCGATTGGATTGTTGGGACAGAGAGGCACCACAACGAGAACTTGACGGTGAGCAGCGTGAATTTCAGCAGCGATGGTAGCTTGGAAGACGACGAGGACTCCCTGGAAAACCAGAACCTTCAGTGGGCTCAGGGAAAAGCAGGGGAGGACCGAGTGCACGTCGTCGTTTCGGAGGAGCACAGCTGGGTATTCGTCGGGATTTACGATGGGTTCAACGGCCCCGATGCTCCTGATTTCTTGCTCTCCAATTTGTACTCGTCCGTGCACAAGGAGCTAAAGGGTCTGTTGTGGGACGACGGGTTTGAATCCCCTACGAACACAATCACTGCCCAACCTGCCTCTTCTTCCCCTGCTTCCCATTCCGATGTCAATTCGAATTCGGAAATGGAAGATTCCGATGGAAATTCGGCGAGGAATCGGTCGGCCGGAGATGCTTGTACTCGGTGCGCAGTTGAGGTGCATGAGAATTTTCCCTGTGCAAGTGGGGACGATGATGGGAAACGAACGAGGGGTAAGAGTTCATCGAAAAGCAGGTACAAGAACAGAGGGTCTGAGAACCAGAGGAGGTGGAAGTGGAGGTGTGAATGGGACCGTGAGCGAGTGGAGCTCGACCGGAGGTTAAAGGAACAGTTACAGAATAGTAGCAATAAATCTGAGTCGGAGGGGTCGAGAGCGATCAACCACTCGGACGTGTTGAAAGCGCTTTCGCAGGCTCTAAGGAAAACAGAGGAGGCCTATTTGGATATTGCGGACAGGATGGTTGTAGAGAATCCGGAGCTGGCGTTGATGGGCTCCTGTGTGCTCGTCATGCTAATGAAGGGTGAGGATGTGTATGTGATGAATGTGGGTGATAGTCGAGCAGTGTTGGCTCAGAAGGCCGAGCCCGATTATTGGTTGGGGAAGATTCGGCAGGATTTGGAGAGAATCAATGAAGAAACTATGCATGATCTGGAAGCGTTGGACGGTGATAGGCCCAGCACGTGTCCGAGTCTGACCGCTTTTCAGCTTAGCGTGGATCATAGCACTAGTGTGGAAGAGGAGGTTCAGAGAATAAAGAGTGAACATCCAGATGATGCTTGTGCTGTGACCAATGATCGTGTGAAGGGTTCCTTGAAGGTCACTCGGGCTTTTGGTGCTGGTTTTCTCAAGCAGCCTAAATGGAACAATGCACTTTTGGAGATGTTCAGAATAGACTACATGGGGAATTCCCCTTACATTAACTGTTTCCCATCTCTATACCACCATAGATTAGGCCCAAAAGACAGGTTTTTGATATTATCCTCTGATGGGCTCTATCAATACTTCACGAATGAAGAAGCTGTTTCTGAAGTTGAACTTTTCATCATGTTCCAACCTGAAGGAGACCCAGCTCAACATCTGGTTGAGGAAGTGCTGTTTCGTGCTGCCAAGAAAGCTGGTATGGACTTCCACGAATTACTCGATATACCACAAGGGGATCGACGGCGTTACCACGATGATGTTTCCATCATTGTTATTTCATTAGAGGGAAGGATATGGAGGTCATGTGTATAA
